# A randomized placebo controlled clinical trial to evaluate the efficacy and safety of minocycline in patients with Angelman syndrome (A-MANECE study)

**DOI:** 10.1186/s13023-018-0891-6

**Published:** 2018-08-20

**Authors:** Belén Ruiz-Antoran, Aranzazu Sancho-López, Rosario Cazorla-Calleja, Luis Fernando López-Pájaro, Ágata Leiva, Gema Iglesias-Escalera, Maria Esperanza Marín-Serrano, Marta Rincón-Ortega, Julián Lara-Herguedas, Teresa Rossignoli-Palomeque, Sara Valiente-Rodríguez, Javier González-Marques, Enriqueta Román-Riechmann, Cristina Avendaño-Solá

**Affiliations:** 1 Department of Clinical Pharmacology, University Hospital Puerta de Hierro Majadahonda, Madrid, Spain; 2 Department of Pediatrics, University Hospital Puerta de Hierro Majadahonda, Madrid, Spain; 3 Department of Neurophysiology, University Hospital Puerta de Hierro Majadahonda, Madrid, Spain; 4 Departamento de Orientación Evaluación psicológica del Colegio Concertado de Educación Especial CEPRI, Madrid, Spain; 5 Department of Cognitive Processes, Universidad Complutense de Madrid, Madrid, Spain; 6 Department of Psychology and Education, Centro Universitario Cardenal Cisneros, Alcalá de Henares, Madrid, Spain; 7 Department of Clinical Pharmacology, IIS Puerta de Hierro-Segovia de Arana, c/ Joaquín Rodrigo, 1, 28222 Madrid, Spain

**Keywords:** Angelman syndrome, Treatment, Minocycline, Developmental changes, Pharmacological intervention

## Abstract

**Background:**

Minocycline is an old tetracycline antibiotic that has shown antiinflammatory and antiapoptotic properties in different neurological disease mouse models. Previous single arm study in humans demonstrated benefits in individuals with Angelman Syndrome (AS); however, its efficacy in patients with Angelman Syndrome has not been assessed in a controlled trial.

This was a randomized, double-blind, placebo-controlled, crossover trial in individuals with AS, aged 6 years to 30 years (*n* = 32, mean age 12 [SD 6·29] years). Participants were randomized to minocycline or placebo for 8 weeks and then switched to the other treatment (a subset of 22 patients) or to receive minocycline for up to 16 weeks (10 patients). After week 16, all patients entered a wash-out 8-week follow-up period.

**Results:**

Thirty-six subjects were screened and 34 were randomized. Thirty two subjects (94·1%) completed at least the first period and all of them completed the full trial. Intention-to-treat analysis demonstrated the lack of significantly greater improvements in the primary outcome, mean changes in age equivalent of the development index of the Merrill-Palmer Revised Scale after minocycline compared with placebo (1·90 ± 3·16 and 2·00 ± 3·28, respectively, *p* = 0·937). Longer treatment duration up to 16 weeks did not result in better treatment outcomes (1·86 ± 3·35 for 8 weeks treatment vs 1·20 ± 5·53 for 16 weeks treatment, p = 0·667). Side effects were not significantly different during minocycline and placebo treatments. No serious adverse events occurred on minocycline.

**Conclusions:**

Minocycline treatment for up to 16 weeks in children and young adults with AS resulted in lack of significant improvements in development indexes compared to placebo treatment. Treatment with minocycline appears safe and well tolerated; even if it cannot be completely ruled out that longer trials might be required for a potential minocycline effect to be expressed, available results and lack of knowledge on the actual mechanism of action do not support this hypothesis.

**Trial registration:**

European Clinical Trial database (EudraCT 2013-002154-67), registered 16th September 2013; US Clinical trials database (NCT02056665), registered 6th February 2014.

**Electronic supplementary material:**

The online version of this article (10.1186/s13023-018-0891-6) contains supplementary material, which is available to authorized users.

## Background

Angelman Syndrome (AS) is a neurodevelopmental disorder characterized by delayed development, intellectual disability, dysmorphic features as prognathism and tongue protrusion, severe speech impairment, seizures, puppet-like ataxic movement, paroxysms of laughter, and abnormal sleep patterns.

For over 20 years it was considered a rare disorder, and although the occurrence of families with affected sibs suggested a genetic aetiology, no known cause could initially be identified. In 1987 Magenis et al. [1] identified a deletion of chromosome 15q11–13 in two patients with AS and subsequent work has shown that AS can be caused by a variety of genetic mechanisms which involve this imprinted region of the genome. All of these mechanisms affect expression of the maternal ubiquitin-protein ligase E3A (UBE3A) gene in the brain. All patients carry at least one copy of paternal UBE3A, which is intact but silenced by a nuclear-localized long non-coding RNA, UBE3A antisense transcript. The absence of the protein product, an E3 ubiquitin ligase, results in the accumulation of regulatory proteins in the post-synaptic density, which is believed to cause abnormal dendritic spine morphology and density in hippocampal pyramidal neurons leading to aberrant synaptic function [2]. These alterations in spine morphology and synaptic function in neurons provides an explanation for the severe profound intellectual disability (ID), lack of speech, difficulties with motor control and planning, significant sleep difficulties, seizures, and unique behavioural features.

There are 4 known aetiologies of AS responsible for the silencing of the UBE3A gene: deletion in chromosome 15q11-q13 (70% of cases), paternal uniparental disomy (UPD; 2% of cases), imprinting defect (3% of cases), and point mutation (10% of cases) [3]. There are 2 documented deletion types classified based on the proximal breakpoint (BP)—class I (BP1-BP3) and class II (BP2- BP3). Class I deletions are bigger, with implications for greater severity in phenotype.

In recent years clearer delineation of the clinical phenotype of AS and improved diagnostic testing has led to improved recognition of the condition and the incidence of AS is now estimated to be between 1 in 10,000 and 1 in 40,000 [4] [5]. Studies of the specific cognitive and behavioural features associated with AS and of the seizure disorder have improved management of the condition and provided insight into the long term outlook for affected patients [6].

Treatment and management is symptomatic with no therapy that addresses the underlying aetiology. A multidisciplinary treatment approach is normally required, relying on appropriate therapies for the physical and neurological problems encountered in this condition, and provisions for special educational needs. Given the very specific cognitive profiles and behavioural features of AS, the treatment should be tailored individually based on the most prominent symptoms. Intensive courses of conductive therapies, similar to those carried out in children with cerebral palsy, have been attempted in AS, with some improvements reported in short-term mobility and communication [7].

Language acquisition is one of the most marked problems in AS. No single communication method works best in AS so every attempt should be made to find a communication system which works for an individual AS child.

Seizures occur in 80–95% of children with AS and usually start in childhood. Seizure types include myoclonic, atypical absence, generalized tonic–clonic, and atonic (“drop”) seizures. Many individuals exhibit multiple seizures types. Seizures usually require broad-spectrum anticonvulsant medication and often combination therapy. The treatment of the epilepsy in AS is often difficult, especially in the early years [8] [9].

Several clinical trials have produced negative results. Attempts to increase transcription from the paternal allele through the use of pro-methylation vitamin supplements did not result in any noticeable improvement. It has been postulated that levodopa/carbidopa and minocycline for its mechanism of action could play a role in therapeutics of AS, but at present evidence supporting its use in clinical practice for AS patients is lacking. Among the reasons postulated to explain these negative findings are thatsome of these treatments do not target the underlying pathophysiology of the disease or that may be the therapeutic attempts are made too late to revert the phenotypic deficits [10].

Minocycline is a semi synthetic tetracycline antibiotic. It is effective against gram-positive and negative infections. Minocycline has been shown to have antioxidant, anti-inflammatory, antiapoptotic and neuroprotective properties in animal models, making it appealing as a potential treatment for neurological disorders [11].

Minocycline has been studied in different neurological disorders, including Fragile X Syndrome (FXS), multiple sclerosis, Alzheimer’s, Parkinson’s disease, stroke, traumatic brain injuries, spinal cord injury, unipolar depression, amyotrophic lateral sclerosis [12–14] and Angelman Syndrome [15]. In Angelman Syndrome, Grieco et al. performed an open label trial in children aged 4–12 years, which showed statistically significant improvementsfollowing 8-week treatment with minocycline in the mean raw scores of the subdomains communication and fine motor ability of the BSID-III (Bayle Scales of Infant and Toddler Development3rd edition), the subdomains auditory comprehension and total language ability of the PLS-IV (Preschool Language Scale 4th edition), the receptive communication subdomain of the VABS-II (Vineland Adaptive Behaviour Scales 2^nd^Edition), and mean scores of the BSID-III self-direction subdomain and CGI scale score. The treatment emergent adverse eventsreported, considered related to minocycline treatment, included lethargy and dizziness. The authors concluded that the administration of minocycline to children with AS is safe and well tolerated, and that minocycline improved the adaptive behaviour of these children suggesting this drug may be an effective treatment for this disorder.

Prior to the publication of study results in medical literature, patients became aware of these results by other means. Expectations and a strong treatment demand emerged among parents and patients associations. This raised the need to conduct a double-blind, placebo controlled clinical trial in order to evaluate the efficacy and safety of minocycline in patients with Angelman Syndrome.

### Objective/hypothesis

The objectives of this study were to determine the efficacy of minocycline on the developmental effects in AS using a randomized, double-blind, placebo-controlled crossover trial. Side effects were closely monitored to assess the tolerability of minocycline treatment. Our hypothesis was that minocycline improves the development index and that it is safe for use in children and young adults with AS.

## Results

### Patient disposition and characteristics

**Figure 1:**
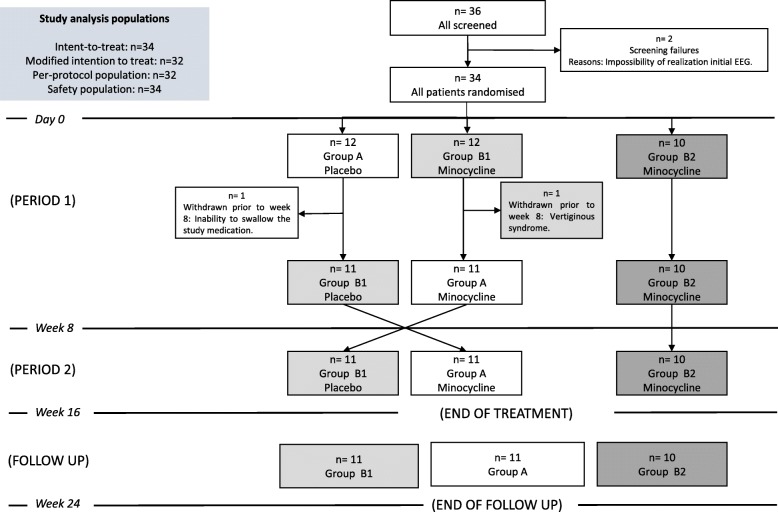
Subject’s disposition

From January 2014 through March 2014, we enrolled 36 consecutive patients. The last patient follow up visit was completed by September 2014.A total of 34 patients were randomly assigned to the trial groups, of them 22 received minocycline (GroupB1 and B2) and 12 received placebo (GroupA) in the initial 8-week period. Two patients did not complete the initial treatment period (one due to inability to swallow the study medication and the other one due to vertiginous syndrome) and were excluded from the efficacy analysis as had not post-baseline assessment (Fig. 1).

A total of 32 patients completed the initial 8-week study period (94.1%) and continued into the additional 8-week period, where patients previously treated with minocycline were to continue on minocycline (GroupB2 = 10 subjects) or switch to placebo (GroupB1 = 11 subjects) in a blinded fashion, and patients previously on placebo were switched to 8-week minocycline (GroupA =11 subjects). All patients completed this second 8-week period, and then entered and completed the final 8-week wash-out period of the trial.

The demographic and disease characteristics at baseline were similar among the two study groups (Table 1).

**Table 1:** Demographic and disease characteristics at baseline according to Trial Group^a^

	PLACEBO (Group A) (*N* = 11)	MINOCYCLINE (Group B) (*N* = 21)
Male sex, *n* (%)	6 (54·5)	10 (47·6)
Age, yr	12·09 ± 6·12	12·48 ± 6·08
Genetic disorder *n* (%)		
Deletion chromosome 15	11 (100)	19 (90·5)
Disomy	0	2 (9·5)
Symptoms		
Seizures, *n* (%)	11 (100)	20 (95·2)
Abnormal sleep	7 (63·6)	3 (14·3)
Concomitant medication use, *n* (%)		
Antiepileptic Drugs	11 (100)	19 (90·5)
Number of antiepileptics/day	1·36 ± 0.50	1.32 ± 0.58
Benzodiazepines/Anxiolytics	6 (54·5)	10 (47·6)
Botulinum toxin	1 (9·1)	4 (19·0)
Melatonin	8 (72·7)	9 (42·9)
Physical therapy and cognitive stimulation^b^		
Behavior therapy, *n* (%)	7 (63·6)	15 (71·4)
Hours per week	2·67 ± 2·10	2·75± 1·75
Logopedic therapy, *n* (%)	8 (72·7)	18 (85·7)
Hours per week	1·64 ± 1·02	2·50 ± 1·24
Physiotherapy, *n* (%)	10 (90·9)	19 (90·5)
Hours per week	1·94± 0·95	2·42± 1·16
Hydrotherapy, *n* (%)	7 (63·6)	12 (57·1)
Hours per week	1·71± 0·48	1·50± 0·67
CGI-S rated by the neuropediatrician, *n* (%)		
1 = Normal	0	0
2 = Borderline mentally ill	0	0
3 = Mildly ill	0	0
4 = Moderately ill	7 (63·6)	14 (66·7)
5 = Markedly ill	4 (36·4)	4 (19·0)
6 = Severely ill	0	3 (14·3)
7 = Most extremely ill patients	0	0

^a^Plus–minus values are mean ± SD. There were no significant differences among the two groups in any of the baseline characteristics

^b^All patients were undergoing special education programs, which contain may differ based on age and on regional policies. Results presented in the table refer to supplementary activities to those local programs

### Efficacy

#### Primary outcome

At week 8, the absolute mean change improvement in the development index of the MP-R Scale (age equivalents in months) from baseline was 2·0 months (SD 3·28) in placebo as compared to 1·9 months (SD 3·16)) in the minocycline treatment groups, leading to non-significant differences between study groups (mean difference 0·095 months, 95% CI -2·22, 2·53, *p* = 0·937) (Table 2).

**Table 2:** Results in the development index of the MP-R Scale at week 8 (primary endpoint) and specific domains of the MP-R Scale

Age equivalents (months)		PLACEBO (Group A)(*N* = 11)	MINOCYCLINE Group B)(*N* = 21)	Absolute mean differences,95% CI
Primary analysis: Absolute mean changes in the DI (age equivalent in months) of the MP-R Scale				
Development index	Baseline	12·55 (6·53)	12·00 (6·27)	0·545, 95% CI (−4·29–5·38)
	Week 8	14·55 (4·90)	13·90 (6·06)	0·641, 95% CI (−3·69–4·97)
	Absolute mean change	2·00 (3·28)	1·90 (3·16)	0·095, 95% CI (−2·22–2·53)
Secondary analysis: Specific domains of the MP-R scale				
Cognition	Baseline	11·55 (6·18)	12·76 (6·89)	−1·216, 95% CI (−6·28–3·85)
	Week 8	15·09 (5·46)	14·24 (5·89)	0·852, 95% CI (−3·50–5·22)
	Absolute mean change	3·54 (4·4)	1·47 (2·22)	2·069, 95% CI (−0·31–4·44)
Fine Motor	Baseline	12·18 (5·94)	11·67 (5·78)	0·515, 95% CI (−3·92–4·95)
	Week 8	14·82 (4·30)	13·71 (5·77)	1·104, 95% CI (−2·94–5·15)
	Absolute mean change	2·63 (4·17)	2·04 (3·93)	0·588, 95% CI (−2·46–3·64)
Visual Motor Coordination	Baseline	12·09 (6·42)	11·29 (6·95)	0·805, 95% CI (−4·34–5·95)
	Week 8	15·55 (4·59)	16·24 (7·48)	- 0·693, 95% CI (−5·75–4·37)
	Absolute mean change	3·45 (4·54)	4·95 (6·31)	−1·497, 95% CI (− 5·89–2·89)
Gross Motor Scale	Baseline	20·64 (7·90)	18·81 (6·83)	1·827, 95% CI (−3·65–7·30)
	Week 8	20·64 (5·87)	20·05 (7·50)	0·589, 95% CI (−4·73–5·91)
	Absolute mean change	0·00 (7·16)	1·23 (3·20)	−1·238, 95% CI (−4·96–2·48)
Socio-emotional	Baseline	19·09 (13·59)	18·57 (7·99)	0·519, 95% CI (−7·24–8·27)
	Week 8	15·82 (7·54)	17·62 (8·26)	-1·801, 95% CI (−7·90–4·30)
	Absolute mean change	−3·27 (7·48)	−0·95 (7·04)	−2·320, 95% CI (− 7·98–3·14)
Adaptive Behavior Scale and Self Care	Baseline	20·73 (8·69)	20·29 (6·55)	0·442, 95% CI (−5·13–6·01)
	Week 8	21·73 (9·64)	21·24 (7·06)	0·489, 95% CI (−5·60–6·58)
	Absolute mean change	1·00 (4·66)	0·95 (3·42)	0·047, 95% CI (−2·90–3·00)

#### Secondary outcomes

##### Week 8 results

Consistently, statistically non-significant differences between placebo and minocycline study groups were observed at week 8 in the changes in relevant subdomains of the MP-R scale, including cognition, fine motor, visual motor coordination, gross motor scale, socio-emotional and adaptive behaviour/self-care domains (Table 2). No changes were observed in any of the categorical domains of the MP-R Scale, i.e. receptive language, infant memory, seep of processing, or expressive language, at 8 weeks.

The proportion of responders (patients with at least 1 month improvement in the age equivalents of the MP-R development index) at week 8 did not show statistically significant differences between placebo (63·6%) and minocycline (66·7%) treatment groups. (Additional file 1: Figure S1).

##### Week 16 results

At week 16, absolute mean changes (SD) from baseline in the development index of the MP-R Scale were 3·00 (3·63), 0·72 (2·56), and 1·20 (5·53), for GroupA, B1 and B2, respectively, and no statistically significant differences were found across the three treatment groups (*p* = 0·401).A comparison between mean changes with 8-week minocycline treatment, regardless of the actual placebo-active treatment sequence (GroupA and GroupB1) vs. 16-week minocycline treatment group (Group B2) was also made and showed no statistically significant differences (1·86 (3·35) vs. 1·20 (5·53), *p* = 0·667) (Table 3). Mean changes following 16-week minocycline treatment were also no significantly different to changes observed right after completing 8-week minocycline treatment (1·20 (5·53) vs. 1·59 (2·89), *p* = 0·675).

**Table 3:** Absolute mean changes in the development index of the MP-R scale at week 16 and Sequential Analysis

Secondary analysis: Absolute mean changes in the DI (age equivalent in months) of the MP-R Scale					
Age equivalents (months)		Group A (Placebo-Minocycline) (*N* = 11)	Group B1 (Minocycline-Placebo) (*N* = 11)	Group B2 (Minocycline-Minocycline) (*N* = 10)	*p* value
Development index	Baseline	12·55 (6·53)	13·09 (6·31)	10·80 (6·33)	0·699
	Week 16	15·55 (5·93)	13·90 (6·65)	12·00 (7·64)	0·495
	Absolute mean change	3·00 (3·63)	0·72 (2·56)	1·20 (5·53)	0·401
Secondary analysis: Development index change after 8 week or 16 week total treatment duration with minocycline					
Age equivalents(months)		Minocycline 8 weeks^a^ (*N* = 22)	Minocycline-16 weeks^b^ (*N* = 10)		Absolute mean differences, 95% CI
Development index	Absolute mean change	1·86 (3·35)	1·20 (5·53)		0·663, 95% CI (−2·55–3·88)
Secondary analysis: Period effect analysis in the cross-over substudy					
		PERIOD 1 (*N* = 22)	PERIOD 2 (N = 22)		Absolute mean differences, 95% CI, *p* value
Development index	Absolute mean change	2·21 (3·37)	− 0·34 (3·24)		2·55, 95% CI (0·80–5·05)

^a^ *Minocycline treatment for 8 weeks include Group A and Group B1 mean changes at week 16 following a total 8-week treatment duration, regardless of the actual placebo-minocycline sequence of treatment;*

^b^ *This corresponds to mean changes at week 16 for Group B2, the subset of 10 patients who received a total of 16 weeks treatment with minocycline*

Twenty two out of 32 patients (11 patients from GroupA plus 11 patients from GroupB1) were included in the cross-over study. Absolute mean changes in the development index of the MP-R scale in patients undergoing 8-week minocycline in two different sequences were: 0·25 (SD 0·72) in patients receiving placebo-minocycline (GroupA) and 1·59 (SD 0·70) in patients receiving minocycline-placebo (GroupB1), with non-significant differences between treatment groups (*p* = 0·20, 95% CI (− 3·45, 0·77). A statistically significant period effect was noticed (*p* = 0·032) (Table 3).

##### Week 24 results

Changes in the development index of the MP-R scale at 24 weeks, including the 8-week wash out period, were compared across the 3 different study groups and showed no statistically significant differences (Group A: 1·30 (SD 4·32); Group B1:-0·09 (SD 3·93); Group B2:-3·00 (SD 5·90), *p* = 0·139), (Table 4). Consistently, statistically non-significant results were observed for the differences between placebo and minocycline study groups in the relevant subdomains of the MP-R scale up to 24 weeks (Additional file 1: Table S1).

**Table 4:** Absolute mean changes in the development index of the MP-R scale at week 24

Age equivalents (months)		Group A (Placebo-Minocycline) (*N* = 11)	Group B1 (Minocycline-Placebo) (N = 11)	Group B2 (Minocycline- Minocycline) (*N* = 10)	*p* value
Primary endpoint: Absolute mean changes in the DI (age equivalent in months) of the MP-R Scale					
Development index	Baseline	12·55 (6·53)	13·09 (6·31)	10·80 (6·33)	0·699
	Week 24	14·80 (6·17)	13·00 (7·89)	7·80 (5·63)	0·689
	Absolute mean change	1·30 (4·32)	−0·09 (3·93)	−3·00 (5·90)	0·139
Secondary analysis: Development index after 8-week wash-out period					
Development index	Absolute mean change	−0·80 (3·39)	−1·00 (3·94)	−4·20 (8·79)	0·365
Age equivalents (months)		After 8 weeks from the suspension of Minocycline (*N* = 21)	After 16 weeks from the suspension of Minocycline (*N* = 11)		Absolute mean differences, 95% CI, *p* value
Development index	Absolute mean change	−0·94 (3·67)	−2·27 (4·12)		−1·32· 95% CI (− 1·65–4·30)

Mean changes in the development index of the MP-R scale following the 8-week wash-out period were − 0·80 (3·39) for GroupA, − 1·0 (3·94) for GroupB1, and − 4·20 (8·79) for GroupB2, with non-significant differences across study groups (*p* = 0·635) (Table 4).

### Parents and clinicians reported outcomes

The severity of the condition was deemed improved by the neuropediatricians (CGI-S) at week 8 in 2 out of 11 (18·2%) patients in placebo and in 6 out of 21 (27·3%) patients in minocycline treated groups, *p* = 0.804. At week 24, improvement in the CGI-S was reported for 1 out of 11 (9·1%) patients in GroupA (placebo-minocycline), 4 out of 11 (36·4%) patients in GroupB1 (minocycline-placebo), and in 3 out of 10 (30%) patients in GroupB2 (minocycline-minocycline), *p* = 0·305.

Differences across study groups in the assessment of the CGI-I rated by neuropediatricians and by parents and show non-significant results at any 8-week (*p* = 0·322 and *p* = 0·972, respectively) and 24 weeks (*p* = 0·116 and *p* = 0·116, respectively). Consistent non-significant results were observed for the proportion of patients with improvements in EEG test at week 8 (*p* = 0·0692) and at week 24 (p = 0·146) (Additional file 1: Table S2).

### Safety

All adverse events reported were treatment emergent adverse events (TEAE). The proportion of patients reporting TEAEs during the initial 8-week period was 8.3% for placebo group and 18.8% for minocycline treatment group (Table 5). Considering both treatment phases, TEAEs reported during placebo or minocycline treatment (regardless of the actual period of study) was 8·7% (2/23) during placebo treatment, 23·3% (eight out of 33 patients) during minocycline 8-week treatment, and 10% (one out of ten patients) for patients who received minocycline for 16 weeks (Additional file 1: Table S3. No SAEs were reported during the study. Treatment emergent adverse events leading to treatment discontinuation occurred just in one patient receiving minocycline and were considered unrelated to study treatment by the clinical investigator. This patient was withdrawn from the study upon parent’s request. No deaths were reported. Treatment emergent adverse events reported include nausea, diarrhoea, constipation, skin alterations, infections and tooth discoloration. All AEs were considered mild and most of them related to study treatment. No changes in laboratory parameters and vital signs were observed during the study.

**Table 5:** TEAEs reporting in each treatment group during the initial 8-week treatment Period 1

	Placebo *N* = 12	Minocyclin 8 week *N* = 22
TEAEs – n° (%) patients	1 (8.3)	4 (18.8)
Number of TEAEs	1	4
Infection	1	
Constipation		
Diarrhea		1
Cutaneous alterations		1
Coloration dental		
Nauseas		1
Edemas		
Increase urine concentration		1
Serious adverse events – n° of patients (%)	0	0
TEAEs leading to treatment discontinuation N (%) patients	0	1 (4.5)^a^

^a^Vertiginous Syndrome.No alterations in laboratory test

## Discussion

In this phase 2 trial involving patients with AS, treatment with minocycline at a dose of 3 mg/kg/day, twice daily orally, resulted in similar absolute mean changes of improvement in the development index (age equivalents in months) of the MP-R Scale at week 8 than placebo (mean 1·9 months vs. 2·0 months, *p* = 0·937). Consistent results were shown for every of the secondary endpoints tested at week 8, including changes in relevant domains of the MP-R Scale, EEG test, and patients/clinicians reported outcomes, for which not even a trend favouring minocycline could be shown. In addition, the responder analysis for the changes in the development index of the MP-R scale and patients/parents CGI reported outcomes showed consistent non-significant results between placebo and minocycline treatments at week 8. Therefore, our study failed to demonstrate the superiority of minocycline over placebo in the treatment of patients with AS.

Consistently, the analysis of patients that crossed-over in the second period shows lack of differences between minocycline and placebo in the mean changes of the development index of the MP-R scale. A significant period effect has been shown; with higher magnitude of changes observed in the first treatment period regardless of the actual treatment received either minocycline or placebo, which might be explained by the expectations from study participants. This is consistent with the higher rates of impression of improvement reported by parents compared to physicians. Thus, observed changes appear mostly related to a study effect than a truly treatment effect as no significant differences between active treatment and placebo could be shown for any of the endpoints analyzed, nor even when comparing different sequences of treatment or active treatment durations. Changes observed may be explained by parent’s and clinician’s expectations, although the contribution of a multidisciplinary intervention during the trial and a potential training effect in the patients cannot be totally ruled out.

It was hypothesized that the 8-week study duration might have been not long enough for the full minocycline effect to be expressed. However, the single arm study conducted by Grieco et al. was able to show significant changes from baseline following 8 weeks treatment with minocycline in patients with AS. Our study was aimed to assess if these encouraging findings were a true treatment effect by including a placebo arm, which addresses the main limitation of the previous trial. Contrary to the previous findings, our study shows that short-term treatment with minocycline is not efficacious in the treatment of AS. The question on whether longer term treatment duration might have been needed remains formally unanswered and the lack of knowledge on the actual mechanism of action of minocycline in the treatment of AS add further uncertainty. However, based on the disappointing results observed in the subset of patients undergoing 16 weeks treatment with minocycline, it is doubted that longer treatment durations may prove to be effective.

This trial was not large enough or of sufficient long-term duration to adequately assess the safety of minocycline in the treatment of AS. However, minocycline belongs to an old class of medicinal products for which an extensive experience of use does exist; making the safety profile of minocycline reasonably well known. Overall, treatment with minocycline was safe and well tolerated.

Our trial has some limitations. The limited sample size and heterogeneity of the studied population precludes drawing firm conclusions on the generalisability of the results to the general AS population and also makes it difficult drawing conclusions in relevant subsets of patients. In addition, longer than 8-week treatment duration might have been needed to express the full potential effect of treatment, but based on previous and current findings in the cohort that followed 16-week treatment, this is very unlikely. Furthermore, despite the control for potential confounding factors, the lack of changes in the rest of educational and therapeutic measures, and the double-blind assessment, an important study effect due to expectations from participants plus a potential training effect could not be prevented. Another weakness is that drug-related side effects have the potential for unblinding both subjects and investigators; for minocycline, these include gastrointestinal, teeth greying and photosensitivity, however there were no significant differences in these effects or any other side effects between the two study groups. We had no episodes of unblinding due to SAEs and only one patient discontinued due to AEs. The addition of a placebo control we aimed to confirm if previous findings were due to a true treatment effect or rather due to a study effect. In addition, the cross-over study design allowed warranting access to active treatment to all patients, making recruitment of subjects less difficult considering that this is a population with a high demand of treatment. It is recognized that this might not be the most appropriate study design to fully explore the potential of minocycline in the treatment of a chronic neurodegenerative condition. Nevertheless, the introduction of a placebo control arm addresses one of the main drawbacks of previous studies in the field and it allows concluding that the previously observed effects cannot be regarded as a short-term benefit due to minocycline in the treatment of AS.

## Conclusions

In conclusion, in this first randomized placebo-controlled study conducted in patients with AS minocycline failed to demonstrate any benefits in the relevant neurological areas affected in AS. These results do not warrant the use of minocycline in AS.

## Methods

From January 2014 through September 2014, we conducted this randomized, double-blind, placebo controlled phase 2 trial at Hospital Universitario Puerta de Hierro Majadahonda. The protocol was approved by the local REC and the national regulatory authority (AEMPS).Written informed consent was obtained from guardians after the procedure(s) had been fully explained. The study was registered at the European Clinical Trial database (EudraCT 2013–002154-67) and with Clinical.Trials.gov (NCT02056665). The study was conducted according to Good Clinical Practice ICH E6 guideline.

### Study design and participants

This was a parallel group, double-blind, placebo-controlled randomized exploratory trial. It was a single centre study, as this is the only reference hospital for AS in our region. The study consisted of an 8-week placebo or minocycline treatment, followed by an additional 8-week treatment period with a cross-over design and a third 8-week withdrawal period. The cross-over design was chosen to facilitate recruitment, by ensuring access to active treatment to all participants.

Eligible patients were child and young adults aged between 6 to 30 years old with a medical diagnosis of AS with molecular confirmation. Patients with history of hypersensitivity to tetracycline, renal and/or hepatic impairment, and any other condition that in the opinion of the investigator was considered clinically relevant and a contraindication for the use of minocycline e.g. uncontrolled seizures, were excluded from participation.

Subjects received experimental treatments on an outpatient basis, either minocycline or placebo, on top of their standard pharmacological and/or non-pharmacological background treatment, which should continue unchanged during the study.

The proposed minocycline dosage was 3 mg/kg/day, twice daily orally, consistent with the dosage tested in the study conducted by Grieco et al. In order to adjust this dosage to the available medicinal product (Aknemin 50 mg capsules), the following weight adjusted doses were used: 100 mg/day for patients < 35 kg (50 mg bid), 150 mg/day for patients between 35 and 50 kg (100 mg-0-50 mg); and 200 mg/day for patients > 50 kg (100 mg bid). The study medication, both active treatment and matched identical placebo, was supplied and labeled by Almirall S.A.

### Randomization and masking

Patients were randomly assigned in a 1:1:1 ratio to receive treatment with placebo for 8 weeks followed by 8-week on minocycline (GroupA), or to receive minocycline for 8 weeks followed by 8-week placebo (GroupB1) or to receive minocycline for 16 weeks (GroupB2). Randomization was performed centrally with the use of a computerized system (Epidat3.1).All study personnel and participants, including the investigators and study-site staff, were masked to treatment assignment.

### Procedures

Patients were assessed on day 1 Visit (baseline), at weeks 8, 16 and 24. After informed consent was signed, subjects fulfilling selection criteria were randomized at day 1 Visit and treatment was started. Physical examination, vital signs, blood samples for clinical chemical and hematologic studies, the Merrill-Palmer Revised Scale of Development, the Clinical Global Impression Scales, and the polygraphic Vídeo-EEG recordings (20 min with a NicoletOne EEGsystem2009 VIASYSHealthcare Inc), were performed at baseline and at every visit thereafter, i.e. at weeks 8,16 and 24. Additionally, adverse events and use of concomitant treatments were recorded throughout the 24 weeks.

The primary outcome was the absolute mean changes from baseline to week 8 in the Development Index (age equivalents in months) of the Merrill-Palmer Revised Scale (MP-R). Secondary endpoints included mean changes in the development index of the MP-R Scale at 16 weeks and 24 weeks, changes in specific subdomains of the MP-R scale at weeks 8, 16 and 24, the proportion of patients with at least 1 month improvement in the age equivalents of the MP-R development index, the rate of improvement in the CGI-S (rated by clinicians) and in the CGI-I (rated by clinicians and parents) at weeks 8,16 and 24. EEG changes at weeks 8, 16 and 24 were evaluated by two independent neurophysiologists, considering changes in background activity, type, number and duration of crisis, widespread tendency to crisis, type of paroxysmal abnormalities recorded and the overall evaluation of the clinical neurophysiologist. Safety and tolerability throughout changes in physical examination, vital signs, laboratory tests and adverse events were also examined.

### Statistical analysis

Demographic and disease characteristics at baseline were presented using descriptive statistics.

The primary and secondary efficacy endpoints were analyzed according to the modified intention to treat principle (mITT), i.e. patients who received study medication and had at least one post-baseline analysis were analyzed according to their original allocation, regardless of the treatment they actually received. For the safety analysis all patients randomized were included and analyzed in the assigned treatment group (ITT).

The primary endpoint analysis was based on the differences in absolute mean changes from baseline to week 8 in the Development index of the MP-R Scale (age equivalents score, in months) between the two main study groups, i.e. patients who received placebo (GroupA) and those who received minocycline (GroupB), using the t-student test.

Similarly, changes in efficacy secondary endpoints at 8-week, 16-week and 24-week were analyzed by t-student test (or ANCOVA where 3 group comparisons were applied, i.e. Group A vs. Group B1 vs. Group B2) for continuous endpoints. Ji square test was used to assess dichotomous variables.

Data from the subset of patients whose sequences included placebo were analyzed by means of mixed models using a standard 2 × 2 cross-over design, taking into account the period, treatment, sequence, and the nested subject within sequence terms.

Sample size calculation was based on the assumption that enrollment of 32 patients (22 subjects in minocycline (Study GroupB) and 10 subjects in placebo (Study GroupA) would provide the trial with 80% power to detect an absolute difference of at least 1·00 unit (1-month age equivalent) in the mean changes from baseline in the development index (age equivalents in months) between minocycline and placebo following 8-week treatment. Due to the absence of published clinical data, the magnitude of the effect considered clinically relevant was chosen upon discussions with experts in the field.

## Additional file

Additional file 1: **Figure S1.** Analysis of responders at week 8, 16 and 24, defined as the proportion of patients with at least 1 month improvement in the age equivalents of the MP-R development index. **Table S1.** Absolute mean changes in specific domains of the MP-R scale at week 24. **Table S2.** Results in CGI-S, CGI-I, EEG test. **Table S3.** TEAEs reported during treatment with minocycline 8-week, minocycline 16-week, or placebo. (DOCX 24 kb)

## Abbreviations

AEMPS: Agencia Española de Medicamentos y Productos Sanitarios
AS: Angelman Syndrome
CGI-I: Clinical Global Impression of Improvement
CGI-S: Clinical Global Impression of Severity
EEG: Electroencephalography
ID: Intellectual Disability
ITT: Intention to treat analysis
MP-R: Merrill-Palmer Revised Scale
SAE: Serious adverse events
SD: Standard Deviation
