# Supplementary material for: A randomized placebo controlled clinical trial to evaluate the efficacy and safety of minocycline in patients with Angelman syndrome (A-MANECE study)
Source: Orphanet J Rare Dis. 2018 Aug 20;13:144. doi: 10.1186/s13023-018-0891-6 (PMC6102900; doi:10.1186/s13023-018-0891-6)
Supplement: Supplementary file 1 — Figure S1. Analysis of responders at week 8, 16 and 24, defined as the proportion of patients with at least 1 month improvement in the age equivalents of the MP-R development index. Table S1. Absolute mean changes in specific domains of the MP-R scale at week 24. Table S2. Results in CGI-S, CGI-I, EEG test. Table S3. TEAEs reported during treatment with minocycline 8-week, minocycline 16-week, or placebo. (DOCX 24 kb) [file 13023_2018_891_MOESM1_ESM.docx]

**Additional file 1**

Figure S1 Analysis of responders at week 8, 16 and 24, defined as the proportion of patients with at least 1 month improvement in the age equivalents of the MP-R development index (attached file)

**Table S1 Absolute mean changes in specific domains of the MP-R scale at week 24**

| **Secondary endpoint: Specific domains of the MP-R scale** | | | |  |  |
| --- | --- | --- | --- | --- | --- |
| **Age equivalents (months)** |  | **Group A**  **(Placebo-Minocycline)**  **(N=11)** | **Group B1**  **(Minocycline-Placebo)**  **(N=11)** | **Group B2**  **(Minocycline- Minocycline)**  **(N=10)** | **p** |
| **Cognition** | Baseline | 11·55 (6·18) | 13·73 (6·69) | 11·70 (6·58) | 0·704 |
|  | Week 24 | 15·10 (6·65) | 13·18 (8·37) | 8·15 (6·90) | 0·112 |
|  | Absolute mean change | 2·60 (6·13) | -0·54 (4·59) | -3·50 (6·80) | 0·085 |
| **Fine Motor** | Baseline | 12·18 (5·94) | 12·82 (5·13) | 10·40 (6·45) | 0·626 |
|  | Week 24 | 13·55 (7·32) | 12·18 (7·97) | 8·40 (4·76) | 0·229 |
|  | Absolute mean change | 1·36 (3·82) | -0·63 (4·96) | -2·00 (5·71) | 0·295 |
| **Visual Motor Coordination** | Baseline | 12·09 (6·42) | 12·09 (6·77) | 10·40 (7·39) | 0·813 |
|  | Week 24 | 13·55 (7·06) | 14·00 (7·32) | 8·60 (5·37) | 0·160 |
|  | Absolute mean change | 1·45 (5·16) | 1·90 (5·08) | -1·80 (7·39) | 0·314 |
| **Gross Motor Scale** | Baseline | 20·64 (7·90) | 20·45 (6·71) | 17·00 (6·84) | 0·492 |
|  | Week 24 | 21·18 (6·46) | 19·73 (7·39) | 18·40 (8·26) | 0·692 |
|  | Absolute mean change | 0·54 (3·23) | -0·72 (6·97) | 1·40 (2·11) | 0·580 |
| **Socio-emotional** | Baseline | 19·09 (13·59) | 18·73 (9·59) | 18· 40 (6·29) | 0·988 |
|  | Week 24 | 13·82 (6·44) | 18·73 (10·44) | 18·80 (11·20) | 0·391 |
|  | Absolute mean change | -5·27 (10·82) | 0·00 (4·93) | 0·40 (11·35) | 0·311 |
| **AdaptativeBehaviuor Scale and Self Care** | Baseline | 20·73 (8·69) | 19·36 (8·10) | 21·30 (4·52) | 0·827 |
|  | Week 24 | 20·00 (6·72) | 22·27 (9,86) | 20·90 (4·70) | 0·775 |
|  | Absolute mean change | -0·73 (4·64) | 2·90 (4·54) | -0·40 (2·31) | 0·084 |

**Table S2 Results in CGI-S, CGI-I, EEG test**

|  | **week 8** | | | | **week 24** | | |
| --- | --- | --- | --- | --- | --- | --- | --- |
| **N (%)**  **p** | Group A  (Pl-Min)  (N=11) | Group B1  (Min-Pl)  (N=11) | Group B2  (Min- Min)  (N=10) | | Group A  (Pl-Min)  (N=11) | Group B1  (Min-Pl)  (N=11) | Group B2  (Min- Min)  (N=10) |
| **Improvement in the CGI-S rated by neuropediatrician** | 2 (18·2) | 3 (27·3) | | 3 (30·0) | 1 (9·1) | 4 (36·4) | 3 (30·0) |
|  | 0·804 | | | | 0·305 | | |
| **Improvement in the CGI-I rated by neuropediatricians** | 4 (36·4) | 2 (18·2) | | 1 (10·0) | 1 (9·1) | 1 (9·1) | 1 (10·0) |
|  | 0·322 | | | | 0·116 | | |
| **Improvement in the CGI-I rated by parents** | 5 (45·5) | 5 (45·5) | | 5 (50·0) | 1 (9·1) | 1 (9·1) | 4 (40·0) |
|  | 0·972 | | | | 0·116 | | |
| **Improvements in EEG test** | 3 (27·3) | 2 (18·2) | | 3 (30·0) | 2 (18·2) | 0 (0·0) | 1 (10·0) |
|  | 0·692 | | | | 0·146 | | |

**Table S3 TEAEs reported during treatment with minocycline 8-week, minocycline 16-week, or placebo.**

|  | Placebo  N=22 | Minocycline 8 week  N=22 | Minocycline 16 week  N=10 |
| --- | --- | --- | --- |
| Averse events – nº of patients (%) | 2 (9.1) | 8 (36.6) | 1 (10.0) |
| Nº. of adverse events | 2 | 9 | 1 |
| Infection | 1 | 1 |  |
| Constipation | 1 | 1 |  |
| Diarrhea |  | 1 |  |
| Cutaneous alterations |  | 2 |  |
| Coloration dental |  | 1 |  |
| Nauseas |  | 2 |  |
| Edemas |  |  | 1 |
| Increase urine concentration |  | 1 |  |
| Serious adverse events – nº of patients (%) | 0 | 0 | - |
| Adverse events leading to discontinuation of regimen– nº of patients (%) | 0 | 1 (4.5)* | 0 |

*Vertiginous Syndrome.No alterations in laboratory test
